# Obstructive Sleep Apnea and Serotoninergic Signalling Pathway: Pathomechanism and Therapeutic Potential

**DOI:** 10.3390/ijms25179427

**Published:** 2024-08-30

**Authors:** Alicja Witkowska, Julia Jaromirska, Agata Gabryelska, Marcin Sochal

**Affiliations:** Department of Sleep Medicine and Metabolic Disorders, Medical University of Lodz, 92-215 Lodz, Poland

**Keywords:** obstructive sleep apnea, serotonin, SERT, sleep disruption

## Abstract

Obstructive Sleep Apnea (OSA) is a disorder characterized by repeated upper airway collapse during sleep, leading to apneas and/or hypopneas, with associated symptoms like intermittent hypoxia and sleep fragmentation. One of the agents contributing to OSA occurrence and development seems to be serotonin (5-HT). Currently, the research focuses on establishing and interlinking OSA pathogenesis and the severity of the disease on the molecular neurotransmitter omnipresent in the human body—serotonin, its pathway, products, receptors, drugs affecting the levels of serotonin, or genetic predisposition. The 5-HT system is associated with numerous physiological processes such as digestion, circulation, sleep, respiration, and muscle tone—all of which are considered factors promoting and influencing the course of OSA because of correlations with comorbid conditions. Comorbidities include obesity, physiological and behavioral disorders as well as cardiovascular diseases. Additionally, both serotonin imbalance and OSA are connected with psychiatric comorbidities, such as depression, anxiety, or cognitive dysfunction. Pharmacological agents that target 5-HT receptors have shown varying degrees of efficacy in reducing the Apnea-Hypopnea Index and improving OSA symptoms. The potential role of the 5-HT signaling pathway in modulating OSA provides a promising avenue for new therapeutic interventions that could accompany the primary treatment of OSA—continuous positive airway pressure. Thus, this review aims to elucidate the complex role of 5-HT and its regulatory mechanisms in OSA pathophysiology, evaluating its potential as a therapeutic target. We also summarize the relationship between 5-HT signaling and various physiological functions, as well as its correlations with comorbid conditions.

## 1. Introduction

Obstructive sleep apnea (OSA) is a breathing disorder characterized by chronic remitting collapse of the upper respiratory tract. The condition results in repeated incidents of apneas and hypopneas and leads to hypoventilation, intermittent hypoxia, hypercapnia, arousals, and sleep fragmentation [1,2,3,4].

The development of OSA is influenced by various factors such as obesity, alterations in the structure of the skull and face, and ventilatory control [5]. OSA diagnosis is established with the usage of polysomnography; its severity is estimated by the apnea-hypopnea index (AHI), and continuous positive airway pressure (CPAP) introduced to upper airways is considered standard treatment [6]. The most prevalent manifestations vary from daytime sleepiness, weariness, intense snoring, and nocturnal awakening. Other potential indicators encompass night sweats, morning headaches, and cognitive impairment [7]. To date, there is an absence of comprehensive reviews addressing the role of serotonin in the pathophysiology of OSA, particularly regarding the translation of this knowledge into clinical practice.

Upper airway patency is evidenced to be preserved by neuromuscular tone, being modulated by neurohormonal agents—one of them being serotonin (5-HT). 5-HT is a monoamine neurotransmitter in the central nervous system (CNS) that acts as a neurohormone and blood factor, influencing cognition, mood, and food intake [8,9]. It also modulates respiratory functions by stimulating hypoglossal motoneurons, enhancing genioglossus muscle activity [10]. In the CNS, 5-HT is regulated by the serotonin transporter (SERT), which controls its reuptake and interactions with receptors [11]. It is also the target of selective serotonin reuptake inhibitors (SSRIs), which are employed in the treatment of disorders such as depression and anxiety [12]. It is also noteworthy that depression and anxiety disorders are more frequently diagnosed in individuals with OSA than in the general population [13]. The pathogenesis of OSA is marked by its complexity, making it challenging to define the mechanisms through which SERT influences the condition precisely. Nonetheless, a genetic predisposition, including polymorphisms of SERT like 5-hydroxytryptamine transporter variable-number tandem repeat (5-HTTVNTR) and 5-hydroxytryptamine transporter gene-linked polymorphic region (5-HTTLPR), has been identified as playing a significant role in its manifestation [14].

Despite the extensive research on OSA, there remains a gap in understanding the nuanced interactions between 5-HT modulation in relation to the pathophysiology of OSA. Consequently, the intricate relationship between 5-HT’s regulatory mechanisms and OSA’s pathophysiology underscores the necessity for further exploration in this domain [15]. Especially since the efficacy of 5-HT modulating medications in OSA depends on the patient’s phenotype. A deeper comprehension of the various 5-HT receptor subtypes and their actions at different locations within the respiratory circuitry is necessary to reevaluate the role of 5-HT modulation in the treatment of OSA. Therefore, in this narrative review, we aimed to summarize knowledge, focus on the dependencies between the serotonergic pathway and OSA, and present up-to-date opinions in this regard with adherence to the clinical implications.

## 2. Materials and Methods

The literature search was performed with the use of online databases, including: “PubMed”, “PubMed Central” and “Scopus” with the search strategy as follows (“Serotonin” OR “5-HT” OR “5HT”) AND (“Sleep Apnea, Obstructive” OR “OSA”). The screening process comprised titles and abstracts. Articles were included in the review upon providing the general aspects explaining the OSA pathogenesis in regard to the 5-HT circulation, concentration, and regulatory mechanisms in both human and animal models.

Although we proposed to conduct a comprehensive review, this paper represents only a regular literature review, not a systemic review performed in accordance with the PRISMA guidelines. Therefore, it is characterized by lower transparency, accuracy, replicability, and a higher risk of biases. Additionally, there was limited data available regarding the associations between 5-HT and OSA, and clinical trials of 5-HT modulating drugs in OSA patients constrain the breadth of insights that can be drawn, thereby limiting the generalizability and depth of the conclusions. To gain a more comprehensive understanding of the research topic, a systematic literature review is necessary to integrate findings from a wider range of studies.

## 3. Serotonergic Pathway and Its Key Role in Physiology and Pathophysiology of the Human Organism

In CNS, the serotonin pathway plays an important role in contributing to neuronal signalization. The pathway includes 5-HT synthesis, 5-HT transport, postsynaptic signal transmission, and 5-HT breakdown. At first, tryptophane, one of the essential amino acids and a serotonin precursor, is converted by tryptophan hydroxylase into 5-hydroxy-L-tryptophan [16]. In the nervous tissue, the mentioned enzyme is encoded by the tryptophan hydroxylase 2 gene [17]. The product of the reaction, 5-hydroxy-L-tryptophan, is then converted into 5-HT by aromatic acid decarboxylase [16]. The transport occurs in secretory granules by vesicular monoamine transporter 1, and then the 5-HT is stored until it is released into the synaptic cleft by presynaptic cell excitation. The substance binds to 5-HT receptors, which are G protein-coupled receptors, leading to G protein-mediated signaling. The only 5-HT receptor that is not a G protein-coupled receptor is the 5-HT_3_ receptor—the ion channel, whose activation results in the cell membrane depolarization [18]. The rest of the receptors transmit the signal via secondary messengers as a part of the excitatory/inhibitory signaling. After achieving it, 5-HT that remains in the synaptic cleft undergoes reuptake into the presynaptic cells via serotonin transporter SERT, the protein encoded by the SLC6A4 gene. This monoamine depends on cytoplasm potassium levels and extracellular sodium and chloride concentrations. Firstly, the sodium ion with serotonin and a chloride ion are bound by the SERT and then transported inside the presynaptic cell again. The remains are again stored in secretory granules or converted by monoamine oxidases to 5-hydroxyindolacetic acid [19].

Melatonin, as a neurohormone released by the pineal gland, is produced from serotonin, initially from tryptophane. The classic pathway after transferring tryptophane into serotonin includes acetylation to N-acetyloserotonin via arylalkylamine N-acetyltransferasethen methylation into melatonin by N-acetylserotonin O-methyltransferase [20]. However, there is also an alternative pathway wherein serotonin is O-methylated into 5-methoxytryptamine and then N-acetylated to melatonin. As melatonin seems to be the main substance involved in circadian rhythm, any disturbance in the serotonergic pathway can directly affect melatonin levels and proper functioning [21]. Bidirectionally, if an external factor, intentionally or not, impairs average healthy sleep architecture or circadian rhythm, it can disrupt melatonin and serotonin levels [22].

5-HT and its receptors are omnipresent in the CNS. Nevertheless, they are mostly located in the brain stem, precisely in the dorsal raphe nucleus (DRN). 5-HT, the major neuromodulator in the CNS, enhances the DRN motoneuron excitability, thus promoting muscle contraction via 5-HT_1A_ and 5-HT2 receptors [23]. What was shown in animal models is that serotonergic receptors in DRN take part in the defensive response associated with anxiety disorder mediation. In addition, DRN 5-HT is chemosensitive, triggering sleep arousal, not enhanced breathing, in case of an increase in carbon dioxide [24]. Additionally, medullary 5-HT neurons are important in homeostatic control by means of breathing regulation, whereas 5-HT found in the hypoglossal nuclei reacts to chronic intermittent hypoxia levels, which can predispose to decompensated OSA [25,26]. 

## 4. The Role of 5-HT in OSA: Genetics, Pathogenesis, Physiological Functions, and Correlations with Coexisting Pathologies

As serotonin is omnipresent in almost the whole human organism, components of its pathway influence every important life function. Any disturbance between the serotonergic system and basic physiological functions can result in disease development and worsening of the general health condition. We chose the most significant ones, which illustrate how intricate the relations are and how widely they can affect human functioning.

### 4.1. Genetics

The dependencies between genetics and the course of the disease may explain variability in studies using serotonergic pharmacological treatment on OSA patients, indicating that not all genotypes benefit from such treatments. OSA pathogenesis might be influenced by certain genetic predispositions. In literature, correlations between OSA and 5-HT receptors, such as SLC6A4, HTR_2C_, HTR_2A_, and HTR_1B_, are strongly associated with OSA and transporter gene polymorphisms [27]. 

According to Schröder et al., the 5-HTTLPR l allele is associated with OSA severity in older patients, which is reflected by the increase of AHI by 4.46 per hour of sleep in comparison to alleles [28]. Including the 5-HT_2A_ receptor, the most common single nucleotide polymorphisms were -1438G/A and T102C in OSA subjects, with -1438G/A polymorphism as a positive risk factor for OSA, especially in men [29]. A similar outcome was exhibited in Piatto et al., pointing to -1438G/A polymorphism to OSA in contrast to also examined T102C [30]. In addition to this, 5-HT_2A_ receptor rs2770304 polymorphism might contribute to the co-occurrence between sleep bruxism and OSA [31], not rs4941573 or rs6313 [32]. A meta-analysis performed by Xu et al. confirmed that polymorphisms in the 5-HTR_2A_ 1438G/A and 5-HTT genes contributed to a greater susceptibility to OSA [33]. Summary of this. Table 1. provides a summary and further elaboration of this information. However, the usefulness of genetic tests in the context of OSA and serotonin pathways is not sufficient to demonstrate any clinical value; the issue requires further research. 

### 4.2. Digestion 

Around 95% of serotonin resides in the bowels and is crucial for initiating peristaltic and secretory reflexes [34]. The blood-brain barrier (BBB), which is impermeable to serotonin, separates central and peripheral serotonergic neurons, but serotonin from the gut can influence the CNS by affecting the BBB’s permeability [35]. Disruption of serotonin levels can lead to digestive disturbances like irritable bowel syndrome and may also be linked to conditions such as Crohn’s disease [36], obesity [37], Parkinson’s disease [38], and depression [39]. Many of the gastrointestinal diseases are associated with an increased risk of OSA and or vice versa [40,41,42]. A disease entity that is particularly significant in the onset and exacerbation of OSA is obesity. Up to 40% of males with a BMI over 30 and 90% of those with a BMI over 40 experience OSA [43]. Obesity pathogenesis might be interlinked with 5-HT, knowing that 5-HT plays a crucial role in food intake regulation and the control of body weight. This notion was further supported by the study by Tecott et al., where 5-HT_2C_ receptor knockout mice were overweight because of abnormal feeding patterns and appetite [44]. Additionally, receptor Agonist Drugs of 5-HT_2C_, such as fenfluramine, sibutramine, and lorcaserin, were used in the preclinical and clinical research and were proven to be effective in reducing body weight. Nevertheless, they were withdrawn by the U.S. Food and Drug Administration because of the adverse effects, including cardiovascular risks [45]. Moreover, OSA might contribute to further weight gain due to factors such as sleep deprivation, daytime drowsiness, and disturbances in metabolism [46]. Table 1. presents a condensed overview and detailed expansion of this information. To date, the research has not delineated distinct connections between OSA, obesity, and 5-HT. The role of obesity-induced endothelial dysfunction also appears to be a viable interlink. Endothelial dysfunction, marked by disrupted hormone production, heightened inflammation, and increased coagulation, stands as a recognized precursor to cardiovascular incidents [47,48]. The precise mechanisms by which OSA compromises endothelium remain elusive. It’s purported that it might be the result of hypoxemia and systemic inflammation [48]. Obesity exacerbates the situation, fostering oxidative stress through various pathways such as lipid peroxidation and reactive oxygen species generation [49]. 

### 4.3. Circulation

The role of 5-HT has been recognized as a potent vasoconstrictive agent in large vessels and a vasodilatory agent in small ones, coordinating the proper blood flow with significant roles in hemostasis and cardiovascular health [50]. Various 5-HT receptor subtypes influence these processes differently, such as 5-HT_1A_ receptors decreasing blood pressure [51], 5-HT_2C_ agonists mitigating 5-HT_2A_ receptor effects [52], 5-HT_3_ antagonists lowering blood pressure [53], and 5-HT_4_ receptors impacting cardiac contractility and potentially contributing to arrhythmias [54]. The matter is particularly important since OSA has been linked to a spectrum of cardiovascular diseases (CVD), encompassing hypertension, arrhythmias, stroke, coronary artery disease (CAD), and metabolic syndrome. The presence of these co-morbidities holds considerable significance, as they are widely recognized to substantially influence healthcare utilization and mortality rates among individuals affected by OSA [55,56]. Associations between OSA, cardiovascular risks, and 5-HT remain elusive, but the recent study by Abdolsamadi et al. shed light on this aspect. It explored the rs6313 and rs6311 polymorphisms in the 5-HT_2A_ receptor gene associated with vasoconstriction, hypertension, and atherosclerosis and its relationship with OSA within the Kurdish population. Their investigation involved 100 OSA patients and 100 healthy individuals, with both groups undergoing polysomnography diagnostic tests. Statistical analysis unveiled significant associations between genotype frequencies of the patient and control groups for rs6311 and rs6313 with OSA, particularly in dominant and codominant models [57]. This information is summarized and expanded upon in Table 1. These findings suggest that both polymorphisms could contribute to OSA susceptibility. However, the study did not find a correlation between the severity of the disease and either of the two polymorphisms.

**Table 1:** Overview of the genetic implications in the OSA pathogenesis regarding susceptibility to OSA, OSA severity, and associations with bruxism, obesity, and cardiovascular risks.

Genetic Implications	Associations and Manifestations
5-HTTLPR l allele	Associations with OSA severity in older patients; manifested by the increase of AHI by 4.46 per hour of sleep in comparison to alleles [28].
5-HT_2A_ receptor polymorphism -1438G/A	Positive risk factor for OSA especially in men [29,30].
5-HT_2A_ receptor polymorphism -rs2770304	Co-occurrence between sleep bruxism and OSA [31].
5-HTR_2A_ polymorphism -1438G/A	Greater susceptibility to OSA development [33].
5-HT_2A_ rs6313 and rs6311 polymorphisms	Associations between vasoconstriction, hypertension, and atherosclerosis and its relationship with OSA within the Kurdish population [57].
5-HT_2C_ receptor knockout in an animal model	Predominant links between weight gain and the pathogenesis of OSA [44].

Abbreviations: serotonin-transporter-linked promoter region (5-HTTLPR), obstructive sleep apnea (OSA), and the apnea-hypopnea index (AHI).

### 4.4. Sleep

The course of OSA is irreversibly bound to changes in sleep structure. Throughout the sleep-wake cycle, 5-HT, noradrenergic, and histaminergic cells display a consistent pattern of activity. They remain active during wakefulness, exhibit decreased discharge rates during non-rapid eye movement sleep (NREM), and either completely cease or significantly reduce their activity during rapid eye movement (REM) sleep to prevent physical enactment of dreams [58,59,60]. Sleep restriction can affect serotonin activity as well. In Davies et al., after occasional sleep deprivation, plasma serotonin along with tryptophan levels was increased as it is the possible explanation for the anti-depressive effect [61], as low serotonin levels are connected with clinical depression [62]. The results are in line with Peñalva et al. and Alfaro-Rodríguez et al., where sleep-deprived rats exhibited increased serotonin levels in neuronal tissue: hippocampus, dorsal raphe nuclei, and suprachiasmatic nuclei [63,64]. Partial (only REM) sleep deprivation can also be therapeutically efficient, as proven in rats [65]. Besides taking part in modulating depression, the serotonergic system can be connected with total and REM sleep deprivation-induced cognitive impairment reflected by memory acquisition and locomotor activity [66]. 

### 4.5. Respiration

Serotonin is a neuromodulator of normal breathing, controlling hypercapnic ventilatory response, not directly baseline ventilation or hypoxic ventilatory response [67]. 5-HT’s imbalance can cause a noticeable change. Exogenously administered 5-HT is evidenced to worsen the respiratory system condition and provoke apneas. Based on the rat’s model, Carley et al. demonstrated that intraperitoneal injection of 5-HT, without the ability to penetrate BBB, resulted in a 2.5-fold increase in the occurrence of spontaneous central apneas during REM without any significant changes in NREM [68]. Meanwhile, the injection of 5-HT into the rabbit’s external carotid artery promptly increased the ventilatory rate in a dose-dependent manner while reducing the tidal volume and integrating phrenic nerve activity [69]. 

Even a slight 5-HT deficiency has a reflection in decreasing ventilatory carbon dioxide sensitivity. However, it can be reversed through a 5-HT increase in the form of SSRI supply [70]. 5-HT receptors connected with respiratory regulation are 5-HT_1A_, 5-HT_2A_, 5-HT_4_, and 5-HT_7_, diminishing or exciting respiratory rhythm depending on the location in the nervous system. In general, endogenous 5-HT facilitates respiratory activity, but with aging, breathing abnormalities associated with 5-HT imbalance aggravate, losing the ability to compensate and defend proper ventilation [71]. Another important factor is gender, as the ability to facilitate response to chronic intermittent hypoxia decreases with age in males but increases in females, pointing to both age and gender’s influence on respiratory functions [72]. Concerning the adult group of people, now widely studied is the mechanism between 5-HT circuits and sudden death in infancy, which can show protective properties in straightening the ability to maintain airflow, facilitating autoresuscitation and gasping, possibly preventing the death of infants [73]. 

Moreover, it is well-established that 5-HT can exert both excitatory and inhibitory effects on medullary respiratory neurons, though the precise mechanisms underlying these effects remain unclear [74]. Studies using an animal model of the newborn rat’s brainstem-spinal cord have shown that 5-HT enhances respiratory rhythm-generating networks in the medulla through 5-HT_1_ receptors and modulates motoneurons via 5-HT_2_ receptors [75]. Furthermore, 5-HT is actively released within the ventral respiratory column (VRC) of the medulla during episodes of hypoxic respiratory depression, underscoring its critical role in respiratory regulation [76]. Additionally, recent research indicates that the excitatory or inhibitory effects of systemic 5-HT administration on respiratory neurons are strongly influenced by the specific expression profiles of 5-HT receptors within these cells. For instance, the presence of Gi-coupled 5-HT_1A_ receptors on glycinergic interneurons in the VRC may account for the paradoxical excitatory effects observed with this typically inhibitory receptor subtype [77]. Subsequent studies demonstrated that the suppression of expiratory neural discharges following stimulation of the raphe obscurus involves both pre- and post-synaptic 5-HT_1A_ receptors [78].

Impaired respiratory processes instigate the defining characteristic of OSA—intermittent hypoxia (IH). IH is defined as bouts of blood O_2_ desaturation and is associated with 5-HT [79]. Studies show hypoxia alters 5-HT levels and receptor functions, with significant reductions in plasma 5-HT and increased uptake and degradation observed in animal models [80]. Hypoxia also affects the 5-HT_1A_ receptor, which is crucial for cognitive functions and can induce phrenic long-term facilitation (pLTF), potentially reducing apneas [81]. Additionally, IH is associated with brain injury and increased apoptosis in the hippocampus and cortex, highlighting the connection between OSA, neurodegenerative conditions, and hypoxia-induced cellular responses [82]. 

These findings suggest that intermittent hypoxia, a key feature of OSA, affects 5-HT levels and receptor function, potentially impacting both respiratory and cognitive processes. Understanding these mechanisms sheds light on the link between OSA and neurodegenerative conditions.

### 4.6. Muscle Tone and Genioglossal Muscle Activity

The most prominent issue in OSA is the collapse of the upper airway, which lacks rigid bony support and relies on surrounding muscles for patency. During sleep, the tone of the upper airway dilator muscles decreases, leading to airway collapse. The upper airway is supported by over 20 dilator muscles, with the genioglossus being particularly important for maintaining airway patency during sleep. The tension of the genioglossus is maintained by the hypoglossal nerve (XII) and modulated by the sleep-wake state, central pattern generators, and chemo/mechanoreceptors [83]. XII nerve electrical stimulation, studied as a potential therapy for OSA, significantly improved objective and subjective measurements of OSA severity by decreasing AHI from 65 to 9 events per hour [84], increasing maximum inspiratory airflow without causing arousals, and showing a 75% success rate over 5 years, as demonstrated in patients with severe OSA [85]. Beyond electrical stimulation, neurohormonal agents, such as 5-HT, also play a role in activating pharyngeal motoneurons, with studies showing its infusion reduces hypoglossal nerve function, affecting airway stability [86]. 

Intake of mianserin, a tetracyclic antidepressant, exhibited a reduced decline in genioglossus muscle activity from NREM to REM sleep and from wakefulness to REM sleep, which can be implemented in OSA patients with airway occlusion due to genioglossus muscle activity impairment [87]. In addition, the intake of 5-HT or histamine diminished a mentioned decrease, pointing to an excitatory influence on genioglossus muscle activity [88]. Conversely, based on the newborn rats’ model, increasing endogenous serotonin levels resulted oppositely, eliciting obstructive events via decreasing genioglossus inspiratory activity. Researchers attribute these findings to the presynaptic action on the axon terminals from the respiratory centers that affect the XII motoneurons [89]. 

These findings highlight the potential of both electrical and neurohormonal interventions and the role of 5-HT in managing OSA. 

## 5. 5-HT in the Context of OSA and Its Correlation with Psychiatric Comorbidities

In recent years, researchers observed a higher prevalence of psychiatric disorders’ symptoms coexisting with OSA, which can be attributed to the elevated scientific interest in the topic. The presence of psychiatric comorbidities in individuals with OSA could impact both their quality of life and their adherence to the CPAP therapy. The notion that sleep fragmentation resulting from OSA causes a higher occurrence of mental disorders and/or their exacerbation is acknowledged globally and supported by a great number of studies [90,91,92].

### 5.1. Depression

The prevalence of depression in individuals with OSA is ranging from 5% to 63%. It’s noteworthy that depression and OSA frequently occur, presenting overlapping symptoms such as difficulty concentrating, loss of memory, emotional fluctuations, and atypical tiredness [92]. This similarity in symptoms makes diagnosing each condition more challenging, as well as understanding the two-way relationship between depression and OSA. However, there are certain speculations of these biomechanisms, including the production of proinflammatory cytokines, neuronal injury, and impaired 5-HT neurotransmission [93,94,95]. 

The pathogenesis of depression has long been linked to 5-HT, with research showing that acute tryptophan depletion can trigger the recurrence of mild depression symptoms in patients who previously responded to 5-HT antidepressants [96]. Moreover, cerebrospinal fluid levels of the primary metabolite of serotonin are often lower in a subset of patients with major depressive disorder, particularly those with suicidal tendencies [97]. 5-HT_4_ receptors, widely expressed in limbic regions like the amygdala and hippocampus, are involved in mood regulation. Polymorphisms in these receptors are linked to unipolar depression, with altered binding and cAMP levels observed in depressed individuals [98]. SERT polymorphisms also play a crucial role in influencing serotonergic function, thereby impacting disease susceptibility and treatment response [99]. SERT deficiency is associated with heightened anxiety and negative behavioral tendencies in adulthood, along with diminished responsiveness to SSRIs [100]. 

**Figure 1:**
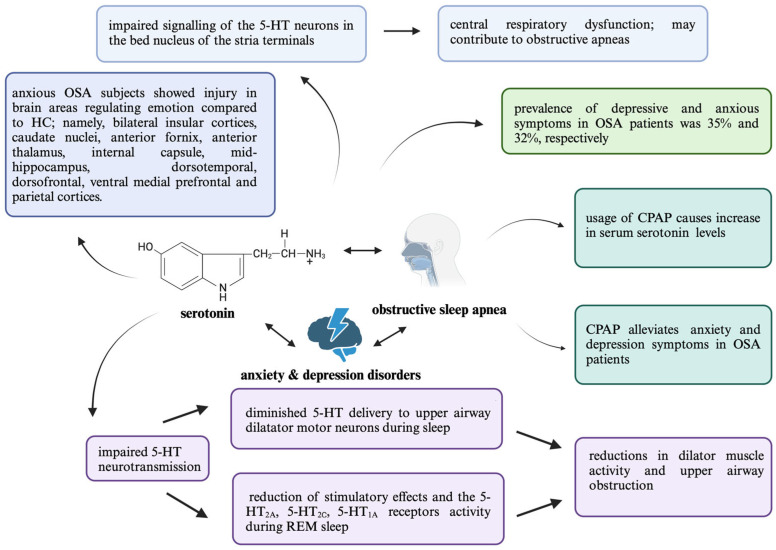
A graphical representation of the associations between 5-HT psychiatric comorbidities—specifically anxiety disorder and depression—within the context of OSA. This aims to highlight the prevalence, the benefits of CPAP usage, and the role of 5-HT neurotransmission. Abbreviations: continuous positive airway pressure (CPAP), obstructive sleep apnea (OSA), healthy controls (HC), serotonin (5-HT).

Much as the role of 5-HT in mood disorders has been extensively studied, its involvement in the pathophysiology of OSA and comorbid depression remains unclear. The plausible codependency might be attributed to the diminished 5-HT delivery to upper airway dilatator motor neurons during sleep. Therefore, it might be associated with sleep-related reductions in dilator muscle activity and upper airway obstruction. In adults, the post-synaptic serotonin receptors responsible for dilation in motor neurons include the 5-HT_2A_ and 5-HT_2C_ subtypes, while the presynaptic serotonin receptor found in motor nuclei is the 5-HT_1B_ subtype, functioning as an inhibitory receptor [101]. Primarily, 5-HT exhibits stimulatory effects on the activity of the 5-HT_2A_ and 5-HT_2C_ receptors located in upper airway motor neurons and 5-HT_1A_ in the respiratory neurons. The receptors’ activity is reduced during REM sleep, which might cause upper respiratory tract collapse [102]. A summary of this information and its expansion can be found in Figure 1. Nevertheless, to the best of our knowledge, no studies are focusing on the dependencies strictly between the 5-HT and depression in the context of OSA’s pathogenesis and course of the disease. 

### 5.2. Anxiety Disorders

Anxiety disorder is a complex, heterogeneous disease entity comprising separation anxiety, selective mutism, phobias, social anxiety disorder, generalized anxiety disorder, and panic disorder. Anxiety disorders have a complex pathogenesis, with 5-HT neurons in the bed nucleus of the stria terminals (BNST) playing a key role [103]. 

Individuals with somatic conditions like cardiovascular, respiratory, such as OSA, or musculoskeletal diseases are at a heightened risk of experiencing anxiety [104]. Rezaeitalab et al. evaluated the prevalence of depression and anxiety in a cross-sectional study of the Iranian OSA population. Based on the Beck Anxiety Inventory (BAI) scoring, 53.9% of the individuals had anxiety. Moreover, OSA severity was associated with the frequency of anxiety, dyspnoea, and sleepiness [105]. A summary of this information and its expansion can be found in Figure 1. Due to overlapping symptoms between anxiety and physical ailments, there is a risk of misdiagnosis, leading to potential under- or over-treatment. Anxiety can worsen the quality of life and treatment outcomes for patients with heart disease, cancer, or lung conditions, and it can increase healthcare costs [106]. Therefore, accurate detection and management of anxiety in patients with physical illnesses are crucial for improving overall patient care. 

### 5.3. Cognitive Dysfunction

Cognitive processes like memory and learning, especially as related to aging and neurodegenerative disorders such as Alzheimer’s disease (AD), are significantly impacted by 5-HT [107,108]. Reduced 5-HT levels and increased 5-HT_1A_ receptor density are linked to accelerated cognitive decline in AD patients. Reductions in 5-HT_2A_ receptor binding were associated with mild cognitive impairment (MCI), as shown by Hasselbach et al. and Smith et al., who observed a significant reduction in SERT availability in MCI patients [24,84,109].

Cognitive performance typically declines with aging due to cumulative damage, including changes in neuronal structures, loss of synapses, and dysfunction in neuronal networks [110]. This decline can be exacerbated by OSA using chronic intermittent hypoxia (CIH). CIH leads to cerebral hypoperfusion, endothelial dysfunction, neuroinflammation, BBB disruption, and cerebral small vessel disease [111]. Sleep fragmentation further worsens these effects by increasing oxidative stress, coagulation, and sympathetic activity [112]. Consequently, individuals with OSA often experience deficits in attention, vigilance, long-term memory, visuospatial abilities, and executive functions, with significant brain damage observed in the prefrontal cortex and notable gray matter loss in the right basolateral amygdala, hippocampus, and the right central insula [113]. 

Interestingly, CPAP therapy improves cognitive performance and neural activity [114]. As reported by Fernandes et al., at baseline, OSA patients had lower β-amyloid_42_ and higher phosphorylated-tau CSF levels than healthy controls, which correlated with reduced brain glucose consumption. Having completed the 12-month CPAP therapy plan, OSA patients demonstrated enhanced cognitive function and a general increase in cerebral ^18^F-FDG uptake [115]. 

**Figure 2:**
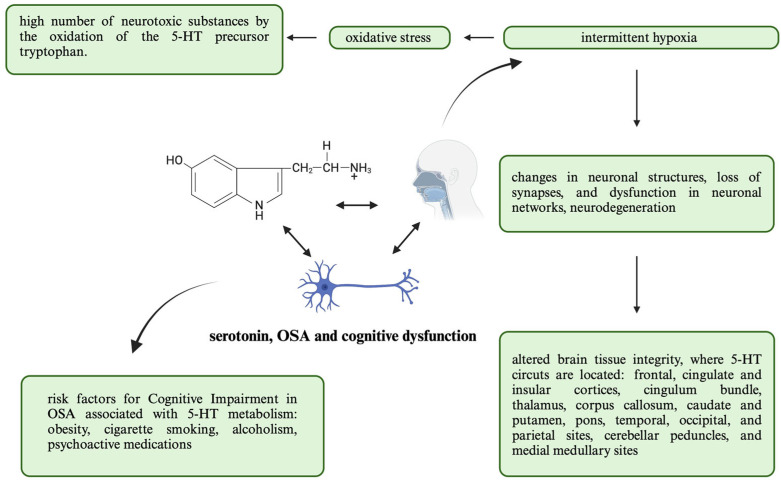
Graphical representation of associations between the 5-HT and psychiatric comorbidities being cognitive impairment within the context of OSA. This aims to highlight the risk factors for cognitive impairment, a consequence of OSA being intermittent hypoxia, and its further implications. Abbreviations: obstructive sleep apnea (OSA), serotonin (5-HT).

Additionally, OSA patients present with an altered brain tissue integrity in the MRI T1-weighted and T2-weighted images compared to controls, including areas such as the frontal, cingulate and insular cortices, cingulum bundle, thalamus, corpus callosum, caudate and putamen, pons, temporal, occipital, and parietal sites, cerebellar peduncles, and medial medullary sites [116]. These areas are particularly important for modulating breathing, sympathetic control, and cognitive functions but are also the locations of the 5-HT circuits [117]. A summary of this information and its expansion can be found in Figure 2.

Currently, there is a lack of specific research linking cognitive impairment caused by OSA to 5-HT levels. However, considering the multifaceted nature of cognitive deficits, this connection is important to explore in future studies. It is also unclear if all cognitive impairments in OSA result from the disease itself, as some may be due to organic brain diseases. 

## 6. Clinical Implications 

To date, there are no guidelines that describe possible pharmacotherapy of OSA. Despite the abundance of scientific evidence, the intricate neural mechanisms and etiology behind upper airway closure in OSA remain elusive, impeding the development of effective pharmacological interventions [118]. With at least 14 serotonin receptor subtypes, serotonergic control of respiration operates at numerous sites within the central and peripheral nervous systems, further complicating efforts to target specific pathways for therapeutic intervention [119]. As previously stated, evidence suggests that agents based on 5-HT transmission may be effective not only in OSA management but also, in particular, in managing comorbid diseases, especially of psychiatric etiology. 

### 6.1. Antidepressants

Sertraline, with high specificity for 5-HT reuptake inhibition [120], was evaluated for sleep-related breathing disorders in a study involving 31 participants over 8 weeks. Initial doses of 50 mg, adjusted to 200 mg daily, were statistically insignificant in the treatment [121]. Study limitations might include the small study group and the differences in the medication dosages. The scarcity of data diminishes the ability to assess the efficacy of OSA’s treatment with sertraline.

Paroxetine, a potent SSRI used for anxiety and phobia, was investigated for its effect on genioglossus muscle activity in OSA patients [122]. In a study by Berry et al., 8 males with severe apnea were administered 40 mg of paroxetine or placebo for 6 weeks. The genioglossus activity increased, but the study showed no significant difference between the paroxetine and placebo groups [123]. Nevertheless, it’s worth emphasizing that the small size of the study group limited the ability to gather comprehensive data. Yet, the correlation between paroxetine and its effect on supplying 5-HT to the motor neurons remains unclear and was not evaluated in other studies. Side effects such as weight gain, a major OSA risk factor, may limit its use in OSA treatment [124]. Especially since obesity is the most important risk factor for OSA, and at least 70% of OSA patients are obese [125]. In some cases, weight loss was considered to be an effective treatment alternative to CPAP for OSA patients, as well as preventing and treating metabolic syndrome [126].

Protriptyline, inhibiting 5-HT and norepinephrine reuptake [127], reduced daytime drowsiness and improved oxygen levels in OSA patients without significant changes in apnea frequency. Additionally, a notable decrease in REM sleep duration and REM apnea episodes was noted. These improvements persisted over six months, accompanied by weight loss, suggesting potential long-term benefits for OSA patients [128]. Therefore, protriptyline might be considered a beneficial agent for patients with REM phenotype in OSA.

Mirtazapine, a 5-HT_1_ agonist and 5-HT_2_/5-HT_3_ antagonist, was evaluated in an animal model of central apnea. It reduced central apnea episodes by over 50% in both NREM and REM sleep in animal studies. In a human trial with 12 OSA patients, 4.5 mg and 15 mg doses of mirtazapine significantly lowered AHI [129,130]. However, side effects like weight gain, a critical OSA risk factor, limit its application despite promising efficacy in reducing AHI and sleep fragmentation [131].

Trazodone, a 5-HT_2_ antagonist, is primarily used as a sedative for insomnia but has shown promise in treating OSA. In a study by Smales et al. involving 15 OSA patients with AHI ≥ 10 events/h during supine NREM sleep, trazodone administration (100 mg) before sleep resulted in a significant reduction in AHI (from 38.7 to 28.5 events/h). Notably, trazodone maintained oxygen saturation levels and did not alter the duration of respiratory events. The reduction in AHI was particularly evident during NREM stage 1 sleep, highlighting trazodone’s potential in reducing sleep fragmentation [132,133]. Its sedative properties, which improve sleep quality, make it a viable candidate for further exploration in OSA management. Furthermore, trazodone is reported to be well tolerated by patients and does not enhance appetite [134], which is particularly important among OSA patients.

### 6.2. Different Medications

Buspirone, a partial agonist of 5-HT_1A_ and low-affinity binder for 5-HT_2_ receptors, has shown potential in treating OSA [135]. A small study by Mendelson et al. found that buspirone reduced the AHI by 36% in a group of five male patients aged 45 on average. Administered at 20 mg, buspirone improved sleep latency and duration without altering mean arterial oxygenation [136]. Buspirone’s affinity for 5-HT receptors is primarily utilized in treating generalized anxiety disorder. Given the high prevalence of anxiety among OSA patients and the known impact of anxiety on prolonging sleep latency, buspirone’s reduction of latency is notable [137,138]. Its lack of sedating effects and absence of respiratory depressant side effects further enhance its suitability for OSA treatment. As a 5-HT_1A_ receptor agonist, buspirone addresses decreased binding and disrupted signaling associated with this receptor, potentially contributing to its efficacy [139]. Notably, this study is the only one evaluating buspirone for OSA, suggesting further research is needed to confirm its potential as a respiratory stimulant during sleep.

Ondansetron is a selective 5-HT_3_ receptor antagonist. Its impact on OSA was examined based on an animal model—an English bulldog, which showed effectiveness in reducing sleep-disordered breathing (SDB). The study group was randomly subdivided and obtained either a placebo, 20 mg, or 40 mg of ondansetron administered orally. Statistical significance was obtained by comparing the effects of ondansetron on SDB in the REM sleep phase. Statistical significance was achieved for the placebo compared with 40 mg of ondansetron [140]. Ondansetron was administered at a dose of 1 mg/kg into the rats’ peritoneal cavity, which demonstrated a notable reduction in REM sleep apneas over the 6-h observation period [141]. To date, research lacks data regarding ondansetron monotherapy instigated in humans. Nevertheless, it might be a valuable agent to be taken into consideration in OSA therapy. Especially since it’s a medication with a safe pharmacological profile with rarely reported adverse effects such as headache (21%), constipation (7%), and abdominal pain (5%), which are non-specific regarding OSA [142]. 

### 6.3. Drugs Combinations

Prasad et al. investigated the combination of ondansetron and fluoxetine. Their role is to stimulate central 5-HT_2_ receptors and inhibit peripheral 5-HT_3_ receptors in the prevention of the development of apnea, regardless of the sleep stage. In total, 35 adults with OSA and AHI > 10 (ranging between 10–98) were included in the prospective, parallel-groups, single-center trial. The study protocol comprised the random assignment of patients into four distinct treatment groups: (1) administration of a placebo either in the morning or evening; (2) a morning placebo coupled with ondansetron (24 mg) in the evening; (3) fluoxetine (5 mg) in the morning and ondansetron (12 mg) in the evening; (4) fluoxetine (10 mg) in the morning followed by ondansetron (24 mg) in the evening. The combination of 10 mg fluoxetine and 24 mg ondansetron resulted in a significant reduction of the AHI by 40.5% from baseline, demonstrating substantial improvement (*p* = 0.005) [143]. 

Hanzel et al. evaluated the influence of a combined therapy consisting of fluoxetine 20 mg and/or protriptyline 10 mg on OSA. The drug combination was chosen upon assessing the effectiveness of fluoxetine in OSA in comparison to another antidepressant, protriptyline. The study protocol differentiated three subgroups: one was administered fluoxetine, another one obtained fluoxetine for the first month of treatment, then discontinued treatment for a week and proceeded with protriptyline treatment, and the third group obtained only protriptyline. The study group consisted of 12 participants—7 males and 5 females, 25–65 years (51 ± 3 years) and bodyweight ranging from 66 to 159 kg (99 ± 7 kg) with the diagnosis of OSA and without any psychiatric conditions. The results obtained were interpreted based on the assumption that both drugs are active in CNS and, therefore, have the potential to modify sleep-related breathing disorders [144]. 

Findings showed that fluoxetine decreased AHI from 57 to 34. At the same time, both medications reduced apneas and/or hypopneas in NREM sleep, fluoxetine from 58 to 32, and protriptyline from 58 to 32. Additionally, a decrease in desaturation episodes per hour of sleep was observed, fluoxetine from 38 to 29 and from 38 to 28 with protriptyline. 

Both drug combinations seem to be a promising alternative to CPAP treatment or could potentially be used as an aid to it, yet further studies are needed to fully evaluate fluoxetine’s potential in reducing AHI. An analysis of adverse events resulting in discontinuation indicates that this drug has a low incidence of serious side effects. It does not induce weight gain as well; in turn, it’s reported that it promotes weight loss at least during the initial 4 weeks of therapy with a mean absolute weight decrease of 0.4 kg [145]. 

The specificity of drugs aimed at 5-HT receptors remains ambiguous, casting doubt on whether these medications also impact other subtypes of 5-HT receptors or different groups of neurons. Furthermore, the varied nature of OSA patients, including their condition’s severity, complicates understanding why certain individuals show positive responses to treatment while others do not. Additionally, several of these drugs come with undesirable side effects, which are the most prominent during the initial stage of instigating pharmacotherapy. Examples of the side effects might include diminished sleep quality, dryness in the mouth, difficulty in urination, slight constipation, decrease in libido, erectile dysfunction, and gastrointestinal disturbances [146]. Moreover, these studies are limited because of small patient groups and the heightened variability in treatment outcomes. It’s noteworthy that SSRI medications exhibit a delayed onset of action, as the initial phase of treatment aims to reduce the activity of 5-HT neurons. Following this initial phase, typically spanning 4–6 weeks, indicates that the time period of the studies might pose another limitation. 

Studies focusing on treating OSA by means of CPAP with aiding combinations of medication targeting the 5-HT receptors would fill the knowledge gap and provide valuable data in this regard.

## 7. Conclusions and Future Directions

As reported, many pathophysiological processes regarding OSA, such as decreased muscle tension, genioglossal activity, and impaired respiratory processes, are directly influenced by 5-HT modulation. Moreover, OSA frequently co-occurs with psychiatric comorbidities, anxiety, and depression, as well as influences and accelerates the neurodegenerative processes. The origin of psychiatric comorbidities is backed by the 5-HT theory, whereas the role of 5-HT in neurodegeneration and cognitive decline is attributed to the loss of the ability to regulate synaptic plasticity and neuronal survival in the adult brain. The 5-HT regulating medications are reported to significantly improve psychiatric comorbidities and alleviate cognitive decline symptoms; interestingly, they might also be effective in OSA management. Recent studies propose several new avenues for investigation, particularly in understanding the interaction between sleep regulation, architecture, 5-HT, and its receptors.

Of all of the evaluated medications, the most promising ones seem to be buspirone because of the observed significant reduction in AHI index and trazodone, with a particularly evident reduction in AHI during NREM stage 1 sleep. One medication that shows potential in aiding OSA is ondansetron. To date, ondansetron has been evaluated on an animal model in combination with fluoxetine, which does not represent the drug’s potential in aiding OSA. Mirtazapine, paroxetine, and protriptyline seem to be helpful in lowering AHI and stimulating genioglossal activity. Nevertheless, the frequently instigated side effect is weight gain, which significantly worsens the disease course and limits its efficacy. 

The obtained results are favorable; on the other hand, it is important to distinguish to which extent the 5-HT modulating medications impact the psychiatric symptoms and to which OSA. The results indicate slight improvements in the degree of hypoxia and AHI reduction, which are promising, and it’s definitely an interesting direction for future research.

More data collected on the larger study groups with standardized protocols is needed to address the interacting effects of 5-HT modulating medications and OSA. The matter is particularly interesting since a significant proportion of patients find it challenging to tolerate the first-line treatment for OSA CPAP. Therefore, developing effective pharmacotherapeutic therapy might be considered a form of active, supportive therapy. Advancements in this area could lead to the development of tailored treatment protocols using pharmacotherapy.
